# Sequential choice vs colonoscopy outreach for colorectal cancer screening: Design and rationale of a pragmatic randomized clinical trial

**DOI:** 10.1016/j.cct.2025.108188

**Published:** 2025-12-16

**Authors:** Shivan J. Mehta, Pamela A. Shaw, Catherine Reitz, Caitlin Brophy, Evelyn Okorie, Keyirah Williams, Abraham Segura, Jinming Tao, Christopher K. Snider, Colin Wollack, Sadie Friday, Katharine A. Rendle, Tamar Klaiman, Karen Glanz, Corinne Rhodes, David A. Asch

**Affiliations:** a Perelman School of Medicine, University of Pennsylvania, United States of America; b Center for Health Care Transformation & Innovation, University of Pennsylvania, United States of America; c Biostatistics Division, Kaiser Permanente Washington Health Research Institute, United States of America; d Penn Center for Cancer Care Innovation, Abramson Cancer Center, University of Pennsylvania, United States of America

**Keywords:** Colorectal cancer screening, Colonoscopy, Fecal immunochemical test, Nudge, Electronic health record, Behavioral economics

## Abstract

**Background:**

Colorectal cancer (CRC) screening rates remain limited, and effective methods for offering the choice of colonoscopy or stool testing through outreach have not been identified. We evaluate the effect of sequential choice compared to colonoscopy outreach on screening completion, and further evaluate behavioral nudges in the electronic health record (EHR).

**Methods:**

In this pragmatic randomized clinical trial, patients were randomly allocated in a 1:2:2 ratio to 1) usual care (no outreach), 2) colonoscopy only, or 3) sequential choice of colonoscopy, then fecal immunochemical testing (FIT). Patients in arms 2 and 3 were additionally randomized to receive either (a) usual care, or (b) a visit-based, clinician-directed nudge facilitated by the EHR with follow-up texting to the patient. The primary outcome is CRC screening completion within 3 years by either colonoscopy, 2 negative fecal immunochemical tests (FIT), or 1 positive FIT followed by colonoscopy within one year.

**Analysis:**

For the patient-directed analysis, the primary outcome will be evaluated by comparing CRC screening completion among patients randomized to either outreach arm (2 or 3) to the no outreach arm (1). We will also compare completion between the colonoscopy only arm (1) and the sequential choice arm (2). For the visit-based analysis, we will compare CRC screening completion among patients between the usual care arms (2a and 3a) and the nudge arms (2b and 3b).

**Conclusion:**

This trial is unique in evaluating the long-term effectiveness of offering sequential choice to colonoscopy alone through a multi-level, centralized outreach and visit-based design.

**Clinical Trials Identifier:**

NCT05693649

## 1. Introduction

Colorectal cancer (CRC) is the second leading cause of cancer death in the United States (US). Routine screening has reduced the burden of disease, but national screening still falls below goals, with rates at approximately 67 %. [1–5] There are also persistent racial, ethnic and socioeconomic disparities in outcomes, particularly among Black patients. [6,7] Multi-level approaches that decrease clinician and patient barriers are important for durably increasing screening rates, including both direct population-based outreach along with clinician recommendations during visits. [8] However, these approach have not been adopted widely and have been difficult to sustain over time.

New insights from behavioral science have helped recognize that humans use systematic shortcuts in thinking that thwart long-term health goals. [9–11] For example, present-time bias reduces the motivation to engage in behaviors whose outcomes are not immediate; status quo bias makes past choices the more likely future choices; and choice overload paralyzes decision making, even when stakes are high. [12–14] However, there is evidence that we can harness these same biases to encourage healthy behaviors. [15,16] Reminders can overcome present-time bias; shifting from opt-in to opt-out framing can increase participating in cancer screening; and both can simplify decisions and prevent overload. [17–21]

Colonoscopy and fecal immunochemical testing (FIT) are evidence-based and top-tier tests for CRC screening. [4,22] FIT achieves a higher response rate in a single outreach, but it requires annual adherence. [23,24] Colonoscopy is more sensitive for identifying polyps and cancer, but the procedure is costly and burdensome for patients. [8] There is evidence that offering a choice of testing can boost screening rates, but there is the potential for choice overload. [25,26] Sequential choice that offers colonoscopy first, then FIT to those who decline or defer has the potential to simplify choice, and it is recommended by clinical guidelines, but there is limited evidence to support this approach. [27–29]

This trial evaluates a multi-level screening intervention that combines direct outreach and nudges in the electronic health record (EHR) during visits to durably increase screening rates. We incorporate behavioral principles such as opt-out framing and reminders while evaluating the comparative effectiveness of sequential choice compared to colonoscopy only.

## 2. Methods

The aims of the 3-year trial are to evaluate the rates of CRC screening completion between patients with 1) no outreach, 2) colonoscopy only, or 3) sequential choice (colonoscopy, then FIT); and to compare the effect of EHR nudges to clinicians and patients during office visits. The trial was approved by the University of Pennsylvania Institutional Review Board and a waiver of consent was granted. [30] It was prospectively registered on clinicaltrials.gov (NCT05693649).

### 2.1. Setting

The trial is being conducted over approximately 3.5 years (June 2023 and January 2027) in 24 primary care clinics within a large, urban, academic health system in the Philadelphia region.

### 2.2. Eligibility and enrollment

Using automated data extraction from the EHR database (Epic Clarity), we identified eligible patients at participating primary care practices within 30 miles of a health system endoscopy site. [31] We included patients aged 50–72 who, at the time of enrollment, were seen by primary care at least once in the past two years, were of average risk of developing CRC, and who were not up-to-date with CRC screening per guidelines at the time. [32] Patients who were not average risk were excluded, including those with a personal or significant family history of CRC, colonic polyps, Hereditary Nonpolyposis Colorectal Cancer Syndrome (HNPCC/Lynch Syndrome), other gastrointestinal cancer, gastrointestinal bleeding, or inflammatory bowel disease (Supplement Table 1). Additionally, patients who may not benefit from screening were excluded, including those with dementia, metastatic cancer, para/quadriplegia, total colectomy, those receiving hospice or palliative care, or those with an elevated chance of mortality within 3 years (Supplement Table 2). Finally, patients were also excluded if they were uninsured, had an active order for FIT (within the last 60 days) or multitarget stool DNA testing (MT-sDNA), or a positive stool test (FIT or MT-sDNA) result in the last 5 years.

We developed a mortality risk indicator to exclude patients who may not benefit from screening. Existing risk indicators included variables such as insurance type that may differentially exclude patients who are already underrepresented in screening but still might benefit. [33,34] Our model uses predictive factors related to end-of-life risk, from domains similar to those represented in the Epic End of Life (EOL) Care Index, with the Medicaid predictor removed, and additional medication, diagnosis, and utilization variables added (Supplement Table 3). This 3-year mortality prediction model was trained on a cohort of 42,451 patients eligible for CRC screening as of 12/1/17, 12/1/18, and 12/1/19 (three years, used to mimic the length of the intervention) using weighted logistic regression with L1 regularization and 5-fold cross validation, which was implemented via LogisticRegressionCV from the scikit-learn library (v1.0.2) in Python. [35] Of this cohort, 779 (1.8 %) patients were deceased within 3 years. The AUC of this prediction model was 0.844 in the held-out test set, performing similarly to the Epic EOL model (0.838). Using a threshold of 0.9, the model’s accuracy was 97.2 % with a positive predictive value of 26.8 %. Upon completion of enrollment, 625 patients of 19,969 eligible patients (3.1 %) were excluded because of an increased risk for mortality within three years.

### 2.3. Randomization

**Fig. 1.**
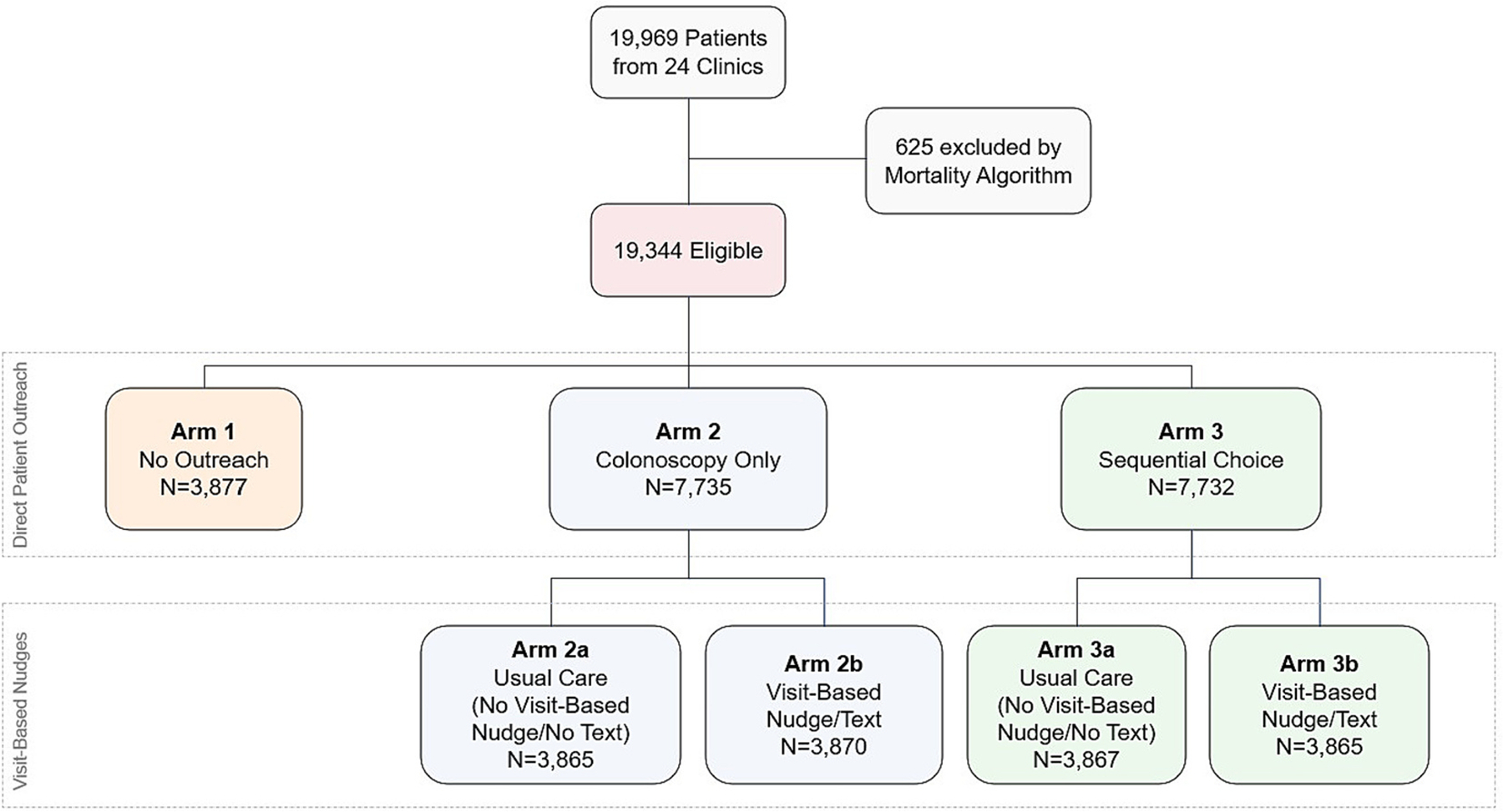
Trial design.

In each of four batches, 5000 eligible patients were randomly selected from the approximately 20,000 eligible patients identified. Patients were individually randomized, stratified by practice, to three arms related to outreach in a 1:2:2 ratio to no outreach (arm 1), colonoscopy only (arm 2), or sequential choice of colonoscopy then FIT (arm 3). In the outreach arms 2 and 3, patients were additionally randomized to receive either (a) no additional intervention, or (b) a visit-based, clinician-directed nudge facilitated by the EHR and follow-up text messaging (Fig. 1). Upon completion of enrollment, 19,344 patients were randomized as follows: arm 1 (no outreach): 3877; arm 2a (colonoscopy only, no nudge): 3865; arm 2b (colonoscopy + visit nudge): 3870; arm 3a (sequential choice): 3867; arm 3b (sequential choice + visit nudge): 3865. See Table 1 for participant characteristics by study arm. Randomization was implemented using block randomization with stratification using Way to Health (W2H), an NIH-funded software platform that automates research and outreach for patient engagement. [36]

### 2.4. Interventions

All patients randomized to an outreach arm (arms 2 and 3) receive centralized direct outreach to complete colonoscopy. Regional physician leads sign bulk order colonoscopy for eligible patients, while the primary care provider (PCP) is listed as the authorizing provider. Patients receive outreach via the electronic patient portal (if active status) or mailed letter informing them their PCP has ordered the procedure, including the colonoscopy order (mailed only) and providing the phone number for direct access to colonoscopy. Outreach is conducted in batches of approximately 4000 patients every 2 months to minimize the burden on clinical services. Additionally, at the time of initial outreach, all patients in arms 2 and 3 receive a text message from the PCP or practice indicating that their primary care provider has already ordered a colonoscopy, with scheduling information. Text messaging via the Way to Health platform is bidirectional, offering automated replies to common colonoscopy related questions. If text messaging is not available, the patient receives similar messaging using Automated Voice Recording (AVR).

Patients who are not up-to-date on screened based on EHR data (Epic Health Maintenance), do not schedule their colonoscopy within 2 months from initial outreach, and have not been ordered for multitarget stool DNA (MT-sDNA) receive an additional mailed and text message reminder. Those in arm 2 (colonoscopy only) receive a mailed reminder about colonoscopy scheduling. In arm 3 (sequential choice), patients receive a mailed FIT kit with instructions, a pre-paid return envelope and a letter describing the stool test as an annual alternative to colonoscopy. The FIT order is signed in bulk by the regional physician lead, with results going back to the PCP as the authorizing provider.

Patients in arms 2 and 3 who have still not completed FIT, or either scheduled or completed colonoscopy at 4 months, receive a final text message reflecting their study arm assignment recommendation. This centralized, direct patient outreach is repeated annually for patients who are not up-to-date on CRC screening, in the same 4 batches of the prior study year.

**Fig. 2.**
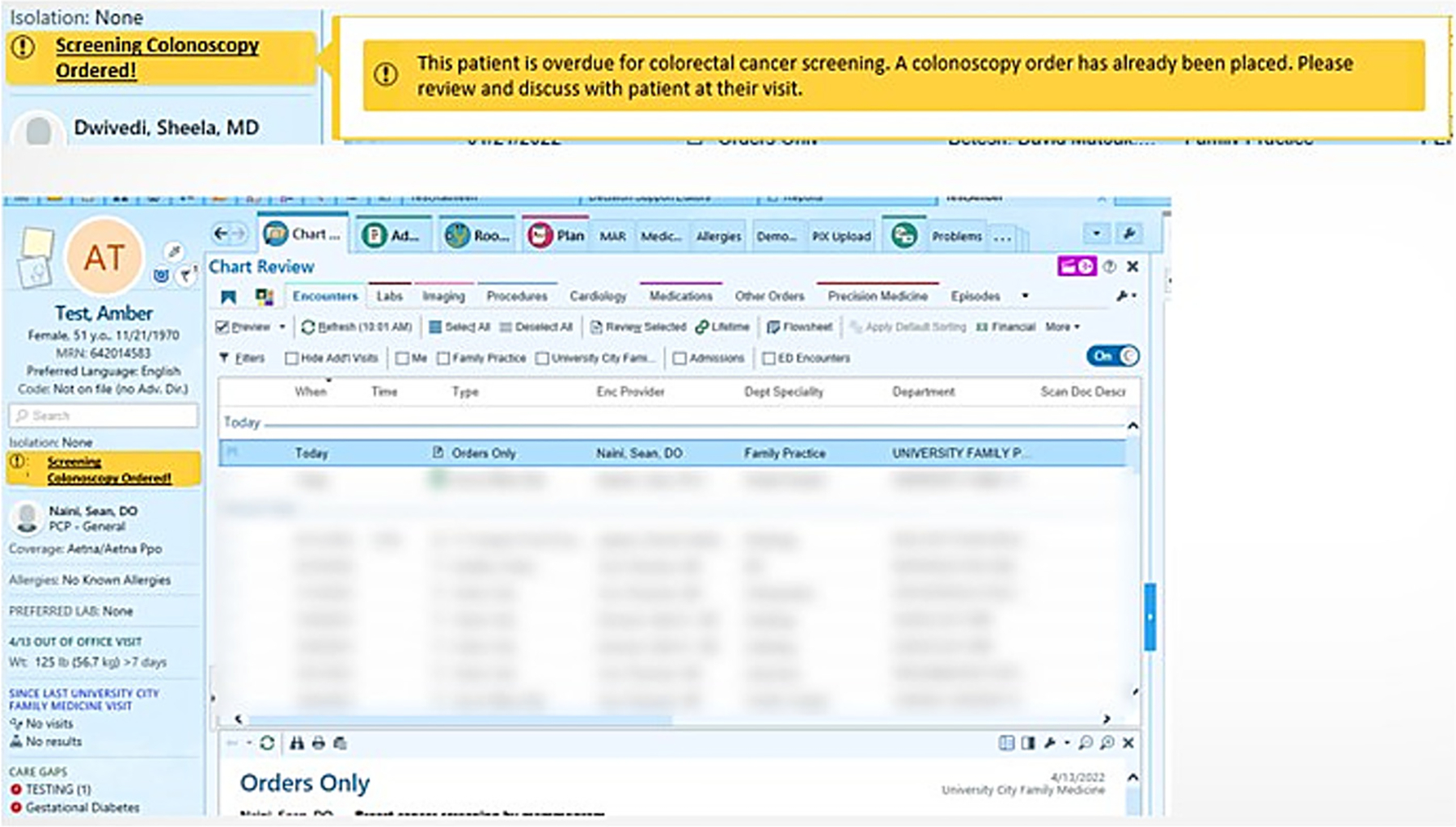
Clinician-facing nudge, storyboard banner (epic).

Patients randomized to a visit-based nudge arm (2B or 3B) who attend a visit with their PCP additionally receive a visit-based, clinician-directed nudge and a follow-up text 3 days post-visit. The nudges include an EHR static highlighted notification (Epic storyboard banner)(Fig. 2) notifying clinical staff (medical assistants, PCPs, and nursing staff) that a colonoscopy and/or FIT order has been placed on their behalf and encouraging discussion around completion. Patients receive a follow-up text message 3 days after the visit reminding them to follow through on active CRC screening orders (colonoscopy, FIT, or both).

Participants randomized to arm 1 (usual care) do not receive any direct outreach or visit-based nudges from this trial. Fig. 1 summarizes the trial design.

As per usual care across the health system, endoscopy reports and follow-up recommendations are provided to patients by the performing gastroenterologist, and patients completing FIT are also be notified of their results. If a FIT positive patient does not schedule diagnostic colonoscopy follow-up within two months of their result and has not received standard follow-up outreach from the practice, the research team sends a note in the EHR to population health coordinators who will provide additional support.

We will obtain baseline data from the EHR on demographics, prior CRC screening, co-morbidities, and mortality risk from automated data extraction and free text searches, as needed. We will use chart review to track outcomes of colonoscopies that are performed with polypectomy, so that we can categorize the finding. Study data are being collected and managed using REDCap electronic data capture tools hosted at the University of Pennsylvania. [37,38]

Changes in Health Maintenance (HM) status are assessed via daily Clarity query to identify documentation of screening results and determine continued outreach. All HM status changes and results (internal or external) picked up by this query are chart-reviewed and only counted toward an outcome if documentation is present in the patient chart.

### 2.5. Mixed methods evaluation

Annually, upon completion of all direct outreach in Batch 3, we send a validated survey guided by the Preventive Health Model (PHM), a selfregulation model shown to predict colorectal screening behaviors in a diverse population, to a select group of enrolled participants. [39,40] The original survey was developed and refined by Vernon and colleagues in 1997 and further validated in a larger and more diverse population by Tiro and colleagues in 2005. [40,41] For a target sample size of 100 completed surveys per year (300 total), and assuming a 20 % response rate, we are randomly selecting a minimum of 500 patients each year, stratified by race (Black patients vs all others) and study arms (intervention arms only) to complete the survey.

The survey consists of 16 individual items spanning five psychosocial constructs: 1) salience and coherence, 2) social influence, 3) cancer worries, 4) perceived susceptibility, and 5) response efficacy. It will also include questions about CRC screening exposure and preferences. Patients randomly selected to complete the survey receive an invite via text message (Way to Health), and participants receive up to 2 automated reminders at 4 and 9 days. If our sample is lower than expected, remaining participants are purposively selected in batches of 10 to receive a manually generated text message offering a scheduled date and time to complete the survey by phone until an appropriate sample is obtained. Survey administration is repeated in Years 2 and 3, with no repeated participants in subsequent years. We monitor responses by study arm, race, and sex to ensure a representative sample.

In year 3 of the trial, a purposive sample of 60 patients will be contacted by phone to conduct qualitative interviews to understand their experience with the specific outreach strategy and the reasons for the effectiveness (or lack of effectiveness) of each intervention. Guided by domains with the PHM, we will also ask semi-structured questions about their perception of the impact and design of outreach.

We will also conduct qualitative interviews with 15 clinicians in the visit-based nudge arms to better understand their experiences with the interventions. Interview participants (patients and clinicians) will be consented and given a $25 gift card in appreciation of their time.

## 3. Analysis

### 3.1. Outcomes

The primary trial outcome is patient completion of CRC screening at 3 years, which can be satisfied by any one of the following: colonoscopy completion at any time, negative FIT completed 2 times, or positive FIT followed by diagnostic colonoscopy within 1 year. The secondary outcomes include CRC screening completion at 3 years by any modality (to include anything satisfying the primary outcome, or completion of sigmoidoscopy, CT colonography, or MT-sDNA); the proportion of time that the patient is adherent to CRC screening process completion (during the 3 years of the study from the date of randomization); and the detection of adenomas, sessile serrated polyps, and advanced neoplasia (adenomas greater than 1 cm, or with high-grade dysplasia or villous histology, or invasive CRC). We will also evaluate the choice of screening test (colonoscopy, FIT, or other modalities) and potential screening harms such as colonoscopy perforation or post-polypectomy bleeding.

### 3.2. Sample size and power

For the patient-directed analysis (Aim 1), after completion of the 3-year time frame for all participants, we will compare CRC screening completion for participants in each of the outreach arms, colonoscopy outreach (arm 2) and sequential outreach (arm 3), separately to the no outreach arm (arm 1); and we will also compare completion between colonoscopy (arm 2) and sequential outreach (arm 3) using intention-to-treat protocol. This includes all patients randomized, such that no evidence of completed CRC screening will be recorded as incomplete and not “missing” regardless of follow-up in the health system. Mortality will be analyzed as a screen failure but is expected to be rare and balanced by study arm. We estimate base CRC screening completion rate of 27 % in arm 1 (no outreach), which is based on estimates of response from a prior study. [29] With 20,000 patients randomly allocated in a 1:2:2 ratio, we will have 85 % power to detect a 3-percentage point increase in response rate of arms 2 and 3 relative to arm 1, and comparing arm 3 to arm 2, accounting for a total of three pairwise comparisons with a two-sided Type 1 error rate of 0.017 (Bonferroni correction of 0.05/3 = 0.017); we will also have 96 % power to detect a 3-percentage point increase in response rate for arm 3 compared to arm 2 (assuming 30 % completion in arm 2 and 33 % completion in arm 3).

For the visit-based analysis (Aim 2), we will compare the primary outcome of CRC screening completion among patients in the direct outreach arms (who have at least 1 visit with the PCP) between the usual care arm (arms 2 A + 3 A) and the visit-based nudge arm (Arms 2B + 3B) that receives clinician directed nudges during the visit and follow-up text messages. Patients in the direct outreach arms (arms 2 and 3) will be randomized in a 1:1 ratio to either receive (A) usual care/no visit-based nudge, or (B) visit-based nudge plus follow-up text. Based on retrospective analysis in this population, we estimate approximately 80 % of patients in each arm will have at least 1 visit during the course of the trial, which translates to 12,800 patients in arms 2 and 3 that will be included in the visit-based analysis (about 6400 in each arm). As this group receives direct outreach, we anticipate a 31.5 % (the average of the estimated completion rate among all patients between Arm 2 and Arm 3) response across the intervention arms for usual care (no visit-based nudges). We will have 80 % power to detect a 2.3 percentage point increase in response rates of arm B relative to arm A.

For our pre-specified subgroup analysis, we estimate 25 % of the study population (5000 patients) will be Black with a baseline completion rate of 23 %, so we will have 80 % power to detect a 5.5 percentage point increase in arms 2 and 3 compared to arm 1, and 80 % power to detect a 4.7 percentage point increase in arm 3 compared to arm 2 (assuming 28.5 % completion in arm 2). For the visit-based nudge comparison and a baseline completion rate of 30.9 % (the average of the estimated completion rate among Black patients between Arm 2 and Arm 3), we estimate that least 80 % of black patients will have at least one visit during the trial (3200 patients), and that we will have 80 % power to detect a 4.7 percentage point increase in arm B compared to arm A (estimate 4000 in arms 2 and 3 in the visit-based analysis).

Power analyses for the difference in proportions were completed in Stata/SE 16.0. While the effects of the individual interventions are seemingly modest, the additional power from the large sample size will help to determine effectiveness of different components in a multi-level intervention, as well as effect on a subgroup of Black patients who have disparities in screening outcomes. Additionally, given the relatively low cost of these interventions, even a small improvement in response could be meaningful when scaled to a large population.

### 3.3. Analytic plan

Analysis will be conducted after completion of the 3-year time frame for all participants according to the pre-specified plan and using intention-to-treat protocol. Mortality will be analyzed as a screen failure but is expected to be rare and balanced by arm. For Aim 1, we will fit a generalized linear model (GLM) for the binomial family with a logit link, robust standard errors, and fixed effects for treatment arm and clinic to compare colonoscopy outreach to no outreach, sequential outreach to no outreach, and sequential outreach to colonoscopy outreach, separately for the primary outcome. [42] We will perform a similar analysis for the detection of adenomas, sessile serrated polyps or advanced adenomas and a zero-inflated Poisson regression model with robust standard errors for the proportion of time covered by screening tests (number of days screened). [43] We will also include socioeconomic and clinical variables in a secondary multivariable model, including age, sex, race, insurance type, income (zip code level), and prior CRC screening, along with interaction terms with the clinician directed EHR nudge assignment. This will allow us to evaluate the effect of patient outreach in the context of visit-based nudges. We will perform a subgroup analysis of Black patients for each of the primary comparisons and conduct additional subgroup analyses for non-rare subgroups in an exploratory fashion, including the same socioeconomic and clinical variables described above for all.

For Aim 2 (visit-based nudge comparison), we will again fit a generalized linear model (GLM) for the binomial family with a logit link, robust standard errors, fixed effect for clinic, and study arm at stage 1 (no outreach, colonoscopy outreach, sequential choice outreach) and stage 2 (nudge, no nudge) to compare visit-based nudges to usual care for the primary outcome. Individuals who attended at least 1 PCP visit will be included in this analysis. As a sensitivity analysis we may consider the potential interaction between the terms for the arm at stage 1 and at stage 2. This will allow us to evaluate the effect of visit-based nudges in the context of colonoscopy and sequential choice outreach. We will perform a similar analysis for the detection of adenomas, sessile serrated polyps or advanced adenomas and a zero-inflated Poisson regression model with robust standard errors for the proportion of time screened (days). We will also include socioeconomic and clinical variables in an exploratory multivariable model, including age, sex, race, insurance type, income (zip code level), and prior CRC screening, along with interaction terms with the patient outreach choice assignment. We will also conduct a sensitivity analysis to examine whether those that showed up for a visit in each arm are similar to those that were originally randomized to that nudge arm, and conduct a reweighted analysis in order to be representative of those originally randomized. We will perform a subgroup analysis of Black patients for each of the primary comparisons and conduct additional subgroup analyses for non-rare subgroups in an exploratory fashion, including the same socioeconomic and clinical variables described above for all.

To evaluate the performance of the 3-year mortality index algorithm, we will perform a 3-year descriptive retrospective evaluation of CRC ordering rates, CRC screening completion rates, number of emergency department visits, number of hospitalizations, and mortality among all patients excluded from participation by the mortality index algorithm.

### 3.4. Mixed methods analysis

The annual embedded survey consists of 16 individual items spanning five psychosocial constructs: 1) salience and coherence, 2) social influence, 3) cancer worries, 4) perceived susceptibility, and 5) response efficacy. [39,40] Each construct can be summed to create a score within each construct, and in total related to beliefs about CRC screening. We will calculate mean differences within each of the five constructs, evaluate each construct on its own scale, and overall look for any associations between CRC screening completion at 3 years and screening beliefs. Finally, we’ll compare screening exposure and preference responses to the type of screening completed at 3 years, if any. We will compare outcomes for those offered the survey and those who were not, to see if receiving the survey was associated with cancer screening completion.

For analysis of the qualitative interview transcripts, two members of the research team will independently review the transcripts for inductive themes through an iterative process using the constant comparative approach. [44–46] After the study team reviews five sets of transcripts, we will develop a coding scheme that will be applied to subsequent data and modified as the study progresses and new themes emerge. The study team members will assess and reassess our inter-rater reliability.

Finally, we will perform a cost analysis using resource utilization to determine the incremental cost of outreach in each arm and the incremental cost of additional patients screened to evaluate resource requirements for scale. We will evaluate the cost from the perspective of the health system, including the time spent by staff that would be needed as part of routine operations when implemented, while not including effort for activities that are only part of the research process.

## 4. Discussion

Durably increasing CRC screening rates equitably among primary care patients requires interventions at multiple levels. By addressing gaps that have been identified from prior research, this trial leverages existing screening infrastructure and layers it with an organized approach, novel use of existing EHR functionality, informed by key concepts from the field of behavioral science.

This will be the first large, prospective trial evaluating the long-term effectiveness of offering sequential choice of colonoscopy then FIT compared to colonoscopy through a multi-level, centralized outreach and visit-based nudge design. Importantly, there is a pre-specified subgroup analysis of Black patients, who are known to have disparities in access and outcomes for CRC. Additionally, if validated, the mortality algorithm could be helpful for the identification of an eligible population without the need for extensive chart review. Further, we have designed and embedded this trial in partnership with health system leadership in a pragmatic design, and have integrated many aspects of the intervention in the EHR and with routine care to make it more efficient and adaptable to other clinical settings.

There are some limitations. While the health system includes a diverse range of primary care practices across the Philadelphia region, the learnings may not translate to other regions or types of health systems. We are not powered to detect differences in clinical outcomes such as cancer incidence and mortality, and we may not be able to fully capture care that happens outside of our health system, despite the integrated EHR and access to some data from other health systems.

In conclusion, this study will provide additional evidence on operationalizing population-level screening programs to reduce CRC burden, and inform future interventions to increase CRC screening.

## Supplementary Material

1

## Figures and Tables

**Table 1:** Participant characteristics.

	Control	Colonoscopy Only	Colonoscopy + Visit Based	Sequential Choice	Sequential Choice + Visit	Total
N	3877	3865	3870	3867	3865	19,344
Age^1^						
Mean (SD)	58.5 (6.6)	58.6 (6.7)	58.4 (6.5)	58.5 (6.6)	58.6 (6.6)	58.5 (6.6)
Sex						
Female	2176 (56.1)	2221 (57.5)	2204 (57.0)	2211 (57.2)	2143 (55.5)	10,955 (56.6)
Male	1701 (43.9)	1644 (42.5)	1666 (43.0)	1656 (42.8)	1722 (44.6)	8389 (43.4)
Race^2^						
Asian	178 (4.6)	173 (4.5)	184 (4.8)	191 (4.9)	203 (5.3)	929 (4.8)
Black	1205 (31.1)	1232 (31.9)	1223 (31.6)	1241 (32.1)	1176 (30.4)	6077 (31.4)
White	2093 (54.0)	2082 (53.9)	2103 (54.3)	2080 (53.8)	2075 (53.7)	10,433 (53.9)
Other	161 (4.2)	159 (4.1)	151 (3.9)	141 (3.7)	179 (4.6)	791 (4.1)
Unknown/Refused	240 (6.2)	219 (5.7)	209 (5.4)	214 (5.5)	232 (6.0)	1114 (5.8)
Ethnicity						
Hispanic	127 (3.3)	3632 (94.0)	3649 (94.3)	3642 (94.2)	3628 (93.9)	18,203 (94.1)
Not Hispanic	3652 (94.2)	120 (3.1)	133 (3.4)	105 (2.7)	132 (3.4)	617 (3.2)
Unknown/Refused	98 (2.5)	113 (2.9)	88 (2.3)	120 (3.1)	105 (2.7)	524 (2.7)
Insurance						
Commercial	2719 (70.1)	2681 (69.4)	2736 (70.7)	2702 (69.9)	2675 (69.2)	13,513 (69.9)
Medicaid	414 (10.7)	403 (10.4)	400 (10.3)	427 (11.1)	406 (10.5)	2051 (10.6)
Medicare	744 (19.2)	781 (20.2)	734 (19.0)	737 (19.1)	784 (20.3)	3780 (19.5)
EMR Portal Status						
Active	3434 (88.6)	3370 (87.2)	3389 (87.6)	3406 (88.1)	3390 (87.7)	16,989 (87.8)
Not Active	443 (11.4)	495 (12.8)	481 (12.4)	461 (11.9)	475 (12.3)	2355 (12.2)
Median Household Income^3^						
Median income (IQR)	$93,588 (55,147–118,207)	$92,593 (55,979–118,443)	$93,588 (54,332–117,331)	$93,103 (54,083–118,207)	$93,588 (55,979–118,207)	$93,588 (55,979–118,207)
Previously Completed CRC Screening^4^						
Any Screening Method	1077 (27.8)	1081 (28.0)	1073 (27.7)	1103 (28.5)	1126 (29.1)	5460 (28.2)

1 Age at time of enrollment.

2 Race is patient self-reported in the EMR; Asian includes Pacific Islander, Other includes American Indian/Alaska Native or self-identified as Other.

3 Income based on median household income by zip code at time of enrollment using the American Community Survey, 2017–2022.

4 Includes the number of unique patients screened by any method prior to trial enrollment, including: FIT, Colonoscopy, Sigmoidoscopy, Cologuard, or CT Colonography.

## Data Availability

No data was used for the research described in the article.

## Appendix A. Supplementary data

Supplementary data to this article can be found online at https://doi.org/10.1016/j.cct.2025.108188.
