# Supplementary material for: Sequential choice vs colonoscopy outreach for colorectal cancer screening: Design and rationale of a pragmatic randomized clinical trial
Source: Contemp Clin Trials. Author manuscript; Available in PMC 2026 Apr 10. (PMC13068070; doi:10.1016/j.cct.2025.108188)
Supplement: 1 [file NIHMS2163388-supplement-1.docx]

**Sequential Choice vs Colonoscopy Outreach for Colorectal Cancer Screening: Design and Rationale of a Pragmatic Randomized Clinical Trial**

Supplementary Material

Supplement Table 1. Summary of ICD-10 codes for exclusion of non-average-risk patients

| **Category** | **ICD-10 Codes** |
| --- | --- |
| Inflammatory Bowel Disease (Crohn’s disease and ulcerative colitis) | K50, K50.0, K50.00, K50.01, K50.011, K50.012, K50.013, K50.014, K50.018, K50.019, K50.1, K50.10, K50.11, K50.111, K50.112, K50.113, K50.114, K50.118, K50.119, K50.8, K50.80, K50.81, K50.811, K50.812, K50.813, K50.814, K50.818, K50.819, K50.9, K50.90, K50.91, K50.911, K50.912, K50.913, K50.914, K50.918, K50.919, K51, K51.0, K51.00, K51.01, K51.011, K51.012, K51.013, K51.014, K51.018, K51.019, K51.2, K51.20, K51.21, K51.211, K51.212, K51.213, K51.214, K51.218, K51.219, K51.3, K51.30, K51.31, K51.311, K51.312, K51.313, K51.314, K51.318, K51.319, K51.8, K51.80, K51.81, K51.811, K51.812, K51.813, K51.814, K51.818, K51.819, K51.9, K51.90, K51.91, K51.911, K51.912, K51.913, K51.914, K51.918, K51.919 |
| GI bleed associated with Inflammatory Bowel Disease | K50.011, K50.111, K50.811, K50.911, K51.011, K51.211, K51.311, K51.811, K51.911 |
| Personal History of Colorectal Cancer | C18, C18.0, C18.1, C18.2, C18.3, C18.4, C18.5, C18.6, C18.7, C18.8, C18.9, C19, C20, C21, C21.0, C21.1, C21.2, C21.8, C78.5, C78.6, C7A.022, C7A.023, C7A.024, C7A.025, D01.0, D01.1, D01.2, D01.3, D37.4, D37.5, D3A.022, D3A.023, D3A.024, D3A.025, Z85.038, Z85.048 |
| Significant Family History of Colorectal Cancer | Z80.0 |
| Colonic Polyps | D12, D12.0, D12.1, D12.2, D12.3, D12.4, D12.5, D12.6, D12.7, D12.8, D12.9, K62.1, K63.5, Z86.010 |

Supplement Table 2. Summary of ICD-10 and CPT codes for patients unable or inappropriate to receive CRC screening based on comorbidities or other health factors

| **Category** | **ICD-10 or CPT Codes** |
| --- | --- |
| **Dementia (ICD-10)** | A81.00, A81.01, F01, F01.5, F01.50, F01.51, F02, F02.8, F02.80, F02.81, F03, F03.9, F03.90, F03.91, F10.27, F10.97, F18.17, F18.27, F18.97, F19.17, F19.27, F19.97, G30, G30.0, G30.1, G30.8, G30.9, G31, G31.0, G31.01, G31.09, G31.1, G31.2, G31.83 |
| **Metastatic Cancer (ICD-10)** | C77, C77.0, C77.1, C77.2, C77.3, C77.4, C77.5, C77.8, C77.9, C78, C78.0, C78.00, C78.01, C78.02, C78.1, C78.2, C78.3, C78.30, C78.39, C78.4, C78.5, C78.6, C78.7, C78.8, C78.80, C78.89, C79, C79.0, C79.00, C79.01, C79.02, C79.1, C79.10, C79.11, C79.19, C79.2, C79.3, C79.31, C79.32, C79.4, C79.40, C79.49, C79.5, C79.51, C79.52, C79.6, C79.60, C79.61, C79.62, C79.63, C79.7, C79.70, C79.71, C79.72, C79.8, C79.81, C79.82, C79.89, C79.9 |
| **Other Gastrointestinal Cancer (ICD-10)** | D01.4, D01.40, D01.49, Z85.038, Z85.048 |
| **Total Colectomy (ICD-10)** | 0DTE0ZZ, 0DTE4ZZ, 0DTE7ZZ, 0DTE8ZZ |
| **Total Colectomy (CPT)** | 44150, 44151, 44155, 44156, 44157, 44158 |
| **Paraplegia/Quadriplegia** | G04.1, G11.4, G13.2, G13.8, G80.0, G82, G82.2, G82.20, G82.21, G82.22, G82.5, G82.50, G82.51, G82.52, G82.53, G82.54, M62.3, R53.2 |

Supplement Table 3. Predictive Factors included in the 3y Mortality Prediction Model

| **Factor** | **Coefficient** |
| --- | --- |
| FRAILTY DX (YN) | -1.312 |
| PRESCRIBED ANTINEOPLASTIC (YN) | 0.855 |
| ALCOHOL RELATED DISORDER DX (YN) | 0.753 |
| NUMBER ADMISSIONS PREVIOUS 1 YEAR | 0.651 |
| PRESCRIBED OPIOID ANALGESIC (YN) | 0.649 |
| PRESCRIBED CARDIOTONIC (YN) | 0.614 |
| ADVANCED ILLNESS DX (YN) | 0.535 |
| HEAD OR NECK CANCER DX (YN) | 0.521 |
| BRONCHUS OR LUNG CANCER DX (YN) | 0.476 |
| PRESCRIBED INJECTABLE ANTIDIABETIC (YN) | 0.380 |
| PRESCRIBED ANTIPSYCHOTIC (YN) | 0.373 |
| PRESCRIBED DIURETIC (YN) | 0.362 |
| CEREBROVASCULAR DISEASE DX (YN) | 0.324 |
| PRESCRIBED PHOSPHOROUS BINDER (YN) | 0.272 |
| COPD AND BRONCHIECTASIS DX (YN) | 0.271 |
| LAST LAB HEMOGLOBIN | -0.242 |
| SUBSTANCE RELATED DISORDER DX (YN) | 0.218 |
| PRESCRIBED POTAASSIUM REMOVING AGENT (YN) | -0.188 |
| PRESCRIBED ORAL ANTIDIABETIC (YN) | 0.167 |
| PRESCRIBED ANTIEMETIC (YN) | 0.166 |
| LAB ALBUMIN MISSING (YN) | -0.162 |
| PRESCRIBED CORTICOSTEROID (YN) | 0.140 |
| NUMBER ED VISITS PREVIOUS 1 YEAR | -0.134 |
| LAB HBA1C MISSING (YN) | -0.125 |
| PRESCRIBED ANTIASTHMATIC (YN) | 0.124 |
| LAB RDW MISSING (YN) | -0.102 |
| LAB HEMOGLOBIN MISSING (YN) | 0.095 |
| CONGESTIVE HEART FAILURE DX (YN) | -0.089 |
| LAST LAB RDW | 0.061 |
| PRESCRIBED GASTROINTESTINAL PROKINETIC AGENT (YN) | 0.060 |
| AGE | 0.028 |
| AGE x FRAILTY DX | 0.027 |
| PRESCRIBED MEDICAL DEVICE | 0.022 |
| AGE x ADVANCED ILLNESS DX | 0.017 |
| HEPATITIS DX (YN) | -0.013 |
| PRESCRIBED LAXATIVE (YN) | 0.008 |
| LAST LAB HBA1C | -0.002 |
| LAST LAB ALBUMIN | 0.000 |

*Weighted Logistic Regression (due to imbalance in outcome) predicting risk of 3-year mortality
Includes L1 regularization and 5-fold cross validation
Modeled on 42,451 patients eligible for CRC screening on 12/1/17, 12/1/18, and 12/1/19; Deceased: 779*
